# Duodenal atresia with apple-peel configuration of the ileum and absent superior mesenteric artery

**DOI:** 10.1186/s12887-016-0690-y

**Published:** 2016-09-05

**Authors:** Radović V. Saša, Lazovic Ranko, Crnogorac Snezana, Banjac Lidija, Suhih Djordje

**Affiliations:** 1 Department of Paediatric Surgery, Clinical Center Montenegro, Children’s Diseases Institute, Ljubljanska bb, Podgorica, 81 000 Montenegro; 2 Department of Digestive Surgery, Clinical Center Montenegro, Ljubljanska bb, Podgorica, 81 000 Montenegro; 3 Clinical Center Montenegro, Clinic of Gynecology and Obstetrics, Ljubljanska bb, Podgorica, 81 000 Montenegro; 4 Department of neonatology, Clinical Center Montenegro, Children’s Diseases Institute, Ljubljanska bb, Podgorica, 81 000 Montenegro

**Keywords:** Apple-peel atresia, Duodenal atresia, Vacular accident

## Abstract

**Background:**

Embryologically, duodenal atresia results from inadequate recanalisation and proliferation of gut epithelius in the 6th week of gestation, while apple-pee atresia of small bowel is a consequence of a vascular accident in subsequent embryonic development, and the two are rather rarely manifested as a joint clinical entity.

**Case presentation:**

We present here a 29 week preterm boy admitted to the intensive care unit due to breathing difficulties and low birthweight. Following clinical, radiographic and ultrasound examination, he was diagnosed with duodenal obstruction and subjected to surgical treatment.

The exploration of abdominal cavity verified duodenal atresia in the second portion with the absence of third and fourth portions of duodenum, superior mesenteric artery, as well as apple-peel atresia of jejunum. Resection of the apple-peel segment of jejunum was done and the continuity of digestive tube was established by the end-to-end duodeno-ileal anastomosis.

**Conslusion:**

This rare case of ours questions the embryology of duodenal atresia suggesting that a mesenteric vascular disruption phenomenon in subsequent embryonic life might be the aetiological factor.

## Background

Duodenal atresia presents a complete intrinsic obstruction of bowels, accounting for nearly half of all atresias affecting duodenum. Incidence of duodenal atresia has been estimated at 1 in 7000 births [1]. The association of duodenal atresias with other congenital anomalies in more than 50 % cases indicates that it is a malformation in early embryonic life [2]. Embryologically, it results from inadequate recanalisation and proliferation of gut epithelium in the 6th week of gestation. Errors in recanalisation occur most frequentlyin the second portion of dudodenum in the area of the ampulla of Vater. Occasionally, duodenal atresia is associated with annular pancreas, and pancreatic tissue surrounds duodenum in theform of aring, causing obstruction [3]. Such aetiology makes duodenal atresias different from other intestinal atresias linked with mesenteric vascular accident and consequent necrosis and resorption of the affected bowel segment [4]. At the cellular level, the development of gastrointestinal tract results from cellular signalisation between the epithelium of embryonic gut of endodermic origin and mesoderm. Sonic hedgehog genes encode members of the Hedgehog family of cell signals. Both are expressed in gut endoderm, whereas target genes are expressed in discretelayers in themesoderm [5]. Due to this significant difference in aetiology, the association of atresia of duodenum with apple-peel atresia of small bowel is very rare. We present here the fourth case of the association of these congenital anomaliesreported in English literature, which queries the embryology of duodenal atresia.

## Case presentation

**Fig. 1:**
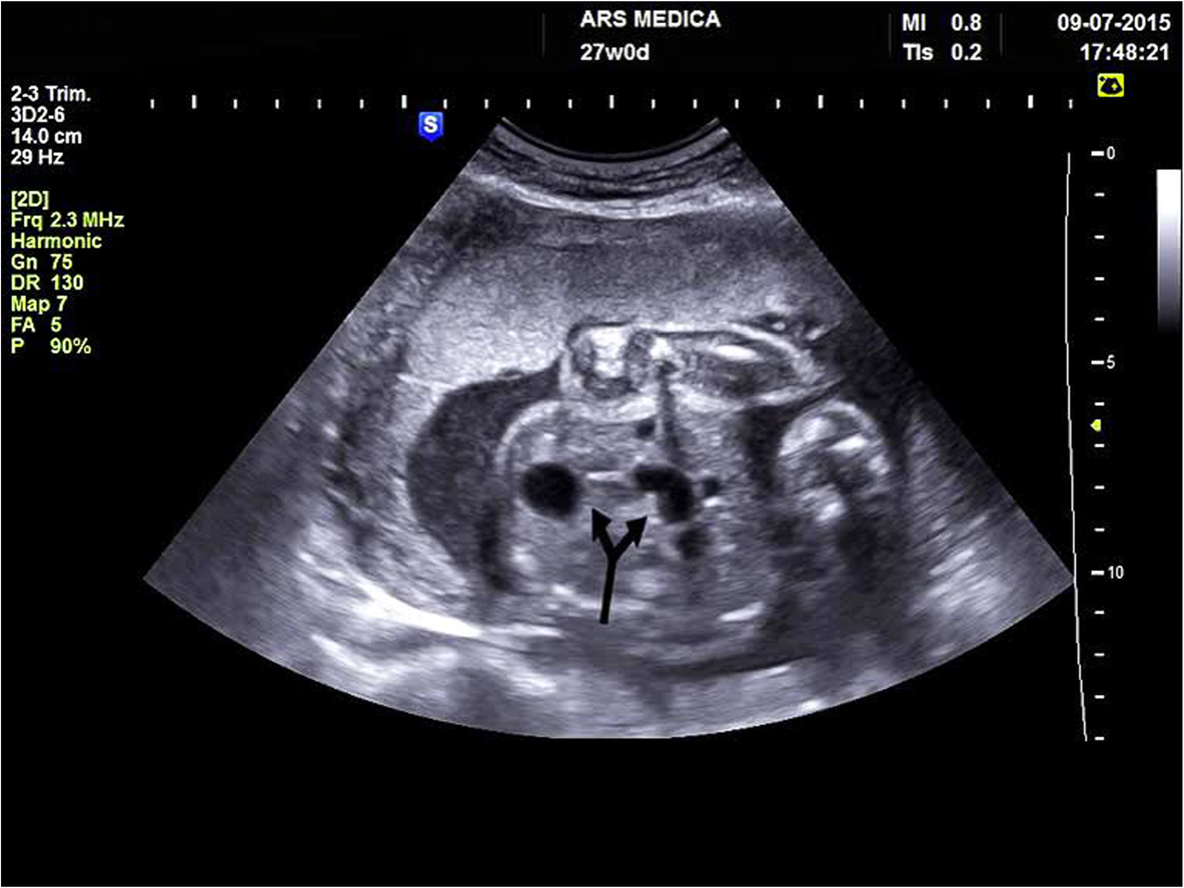
Antenatal ultrasonography 27 th week GA revealed several hypoechoic formations corresponding to dilated stomach and small bowel

**Fig. 2:**
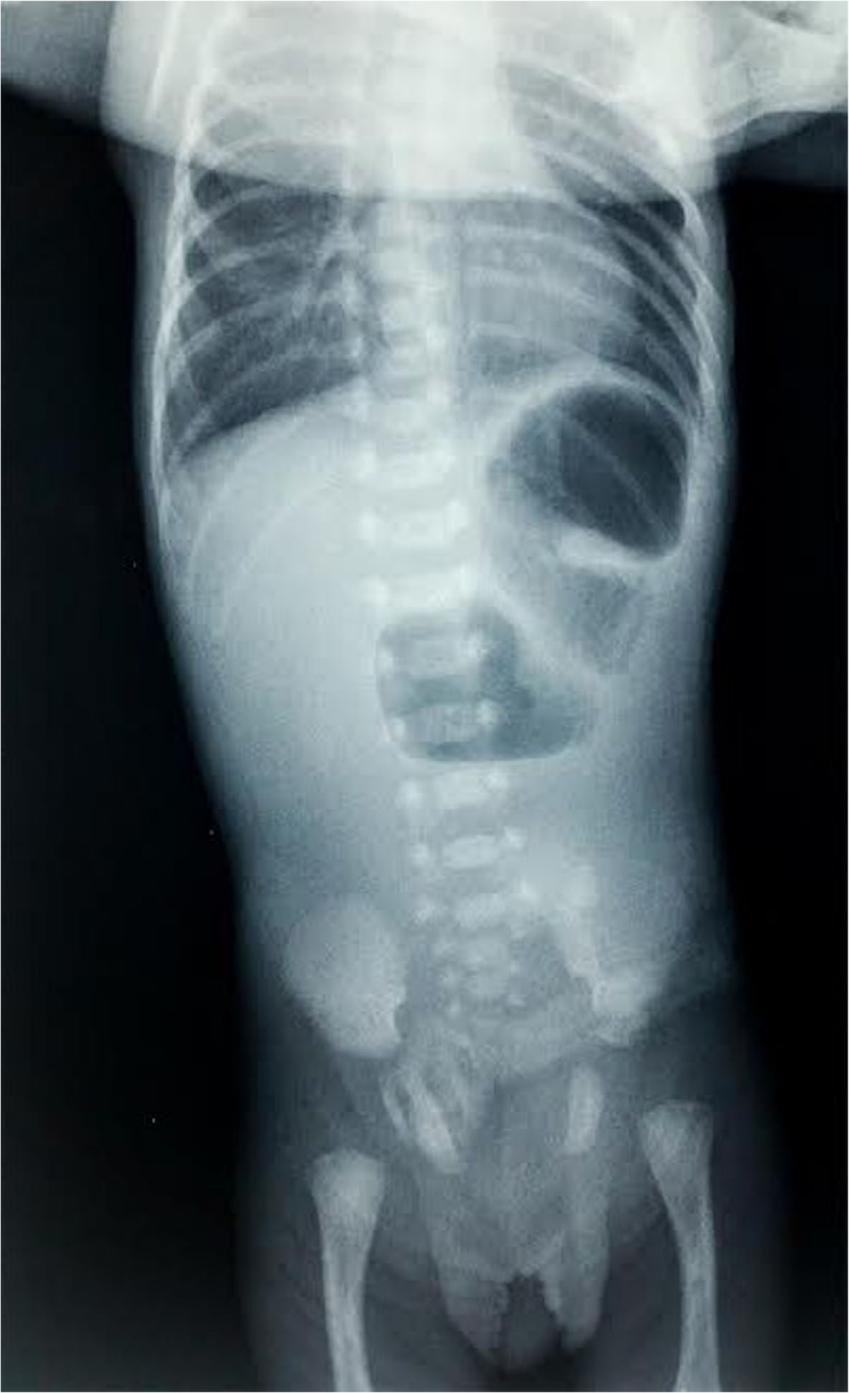
X-ray flat plate abdomen-showing clasical double-bubble apperance of duodenal atresia

**Fig. 3:**
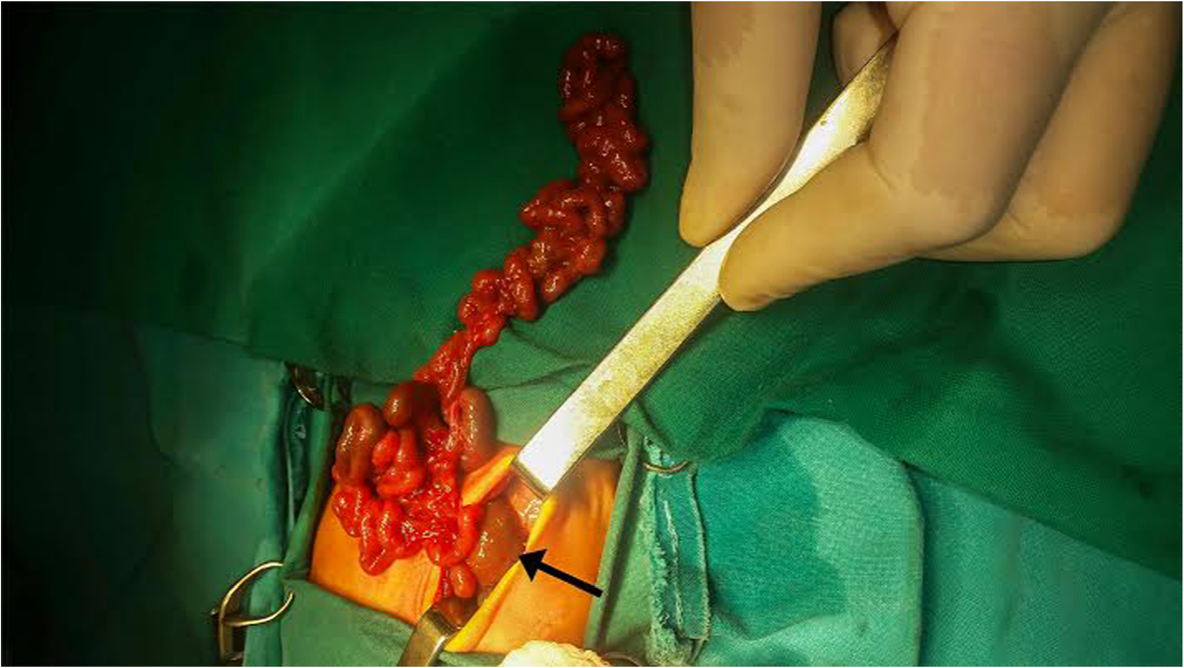
Dilated duodenum and apple-peel jejunal atresia

A two-day old preterm boy (29 weeks GA),weighing 1.240 kg, Apgar score 5/6, was referred from the maternity ward to the intensive care unit for breathing difficulties and low birthweight. Antenatal ultrasonography of abdomen in 27th week GA revealed several hypoechoic formationscorresponding to dilated stomach and small bowel. The quantity of amniotic fluid was normal with minor hyperechoic changes that may have corresponded to the presence of blood in amniotic fluid (Fig. 1). Cytogenetic analysis of amniotic fluid established the presence of a normal chromosome set for male sex. Upon admission, the male infantshowed tachydyspnea, his breathing was irregular, he was cyanotic with livid acrama, displayed reduced spontaneous activity and provoked reactivity, feeble cry, discontent, occasional groaning. The abdomen was below the level of the thorax, soft, mobile on respiration. External genitalia was typical for male gender and anal opening was normally situated. There were no deformities of extremities. The tonus was lower, the activity slowed, reflexes were displayed. Due to respiratory instability upon admission, he was intubated and kept on ventilator support. Nasogastric tube was placed and drained bilious aspirate. Plain radiograph of the abdomen showed the classic dilatation of the proximal duodenum and stomach with total absence of distal bowel gas (Fig. 2). Echocardiographic and clinical findings of the heart were within the physiological limits. Following the initial stabilisation, the right transversal supraumbilical laparotomy was used to access the abdominal cavity. A verification was made of an extremely dilated atretic part of the second portion of duodenum with the absence of the third and fourth portion of duodenum and superior mesenteric artery, as well as the apple–peelconfiguration of the remaining small bowel that isvascularised by arteriaileocecalis (Fig. 3). Continuity apple-peel and jejunum after incision was checked with orogastrictubesize 4 withthe attempt of passage of saline solution. Elimination of the solution was slowed with the absence ofintestinesdilatation, hence the frozen section biopsy of full thickness of extremely thin wall of the intestine was suggested. This was the material for histopathology analysisfollowed by absence of ganglion cells.

Resection of the jejunum and initial portion of the ileum was done up to the vascularisation level of arteria ileocecalis, while preserving cca 10 cm of terminal ileum and ileocecal valve that indicated microscopically normal bowel morphology. Transversal incision on the dilated duodenum was used to access the lumen and to identify by the back wall hyperemic Papilla Vateri, from which bilious aspirate was drained by the manual stimulation of thenormally positioned gallbladder. The continuity of digestive tube was established by the end-to-end duodeno- ileal anastomosis by using polyglactin absorbable suture 6. 0 as sewing material. A size 6 transanastomotic silicone orogastric tube was placed. Postoperatively, the baby was kept on ventilator support. He succumbed on the third postoperative day due to cardiorespiratory failure. The autopsy confirmed the clinical cause of death through the presence of hyaline membranes in alveoli, as well as preserved continuity of bowel anastomosis and absence intestinal contents leakage.

## Discussion

Duodenal atresias and stenoses are most frequent in relation to distal parts of small bowel. By prenatal ultrasonography, it is possible to establish the diagnosis among 52 % patients. Duodenal atresia is manifested by a“double-bubble” sign, in the way that the first bubble corresponds to the stomach, while the second one matches postpiloric prestenotic dilated duodenum [5]. Half of the infants have birthweight below 2.5 kg, which correlates to hydramnion because fetus is not able to absorb nutrients from the amniotic fluid. Clinically, there is manifestation of symptoms and signs of high bowel obstruction and presence of bilious vomiting several hours after delivery. Among 15 % infants, the obstruction is above the ampulla of Vater, which results in the absence of bilious vomiting [6]. In 25–40 % cases, the anomaly is associated with trisomy 21 (Down syndrome, which suggests possible genetic origin) [7]. The absence of associated congenital anomalies in our patient suggests an error in later embryonic development. Apple-peel atresia is a rare form of intestinal atresia of duodenum associated with small bowel spiraled around arteria ileocecalis as peeled apple skin. It results from a vascular accident affecting small bowel thus irrigated by arteria mesenterica superior. Vitality is preserved by ileum that, by way of anastomoses, gains irrigation from arteria colicae mediae through arteria ileocecalis [8]. Our patient had duodenal atresia related to the second portion of duodenum with the absence of the third and fourth portions of duodenum and arteria mesentericae superior, as well as a consequent apple-peel atresia of jejunum, which is the fourth case of the association of these two forms of atresia reported in literature. The length of the affected jejunum and segment of ileum in our patient suggests a vascular accident of the main trunk of arteria mesentericae superior, in contrast to the reported cases of the affected segment of apple-peel jejunum being shorter as a result of the obstruction of its branch [9, 10]. Naturally, separate effects of two different aetiological factors cannot be excluded as a fact either, but the absence of associated anomalies strongly suggests vascular accident in the process of its development.

## Conclusion

Duodenal atresia associated with the apple-peel configuration of small bowel is a very rare anomaly. Its onset cannot be accounted for by the absence of recanalisation in the 6th week of embryonic development, but the case presented here indicates that mesenteric vascular accident be the aetiological factor.
